# A Comparative Analysis of Impact of Universal Two-Child Policy on Maternity Insurance Fund in Jiangsu Province and Guangxi Zhuang AR

**DOI:** 10.3390/healthcare9040468

**Published:** 2021-04-15

**Authors:** Henry Asante Antwi, Lulin Zhou, Xinglong Xu, Tehzeeb Mustafa

**Affiliations:** 1 Centre for Health and Public Policy Research, Jiangsu University, 301 Xuefu Road, Zhenjiang 212013, China; lulinzhou@yahoo.com (L.Z.); 5103140204@stmail.ujs.edu.cn (T.M.); 2 School of Management, Jiangsu University, 301 Xuefu Road, Zhenjiang 212013, China; 1000004932@ujs.edu.cn

**Keywords:** maternity, insurance, health, security

## Abstract

The maternity insurance fund in some provinces in China has accumulated unprecedented deficit levels. This imminent depletion can cause a catastrophic health crisis for maternal health. This study analyzed the post-policy impact of key factors on maternity insurance income inflow and outflow in Jiangsu Province and Guangxi Zhuang Autonomous Region (AR). We applied Pasera’s ARLD model and VECM Granger Causality Test to establish long- and short-term impact of selected factors that determines the income and expenditure of the maternity insurance fund in the two regions based on data from 2011 to 2019. Our results show that the addition of new births due to the universal two-child policy has increased the per capita utilization of the maternity insurance fund in both areas. We further observed that the impact of the maternity insurance contribution rate to the maternity insurance fund decays with time giving a long-run limited impact in both provinces. Thus the positive impact is stronger in the short term, but in the long term, its influence or contribution to stability of the funds reduces. The positive impact of interest from investment in the maternity insurance fund is however insignificant in both provinces, giving a major cause for concern on its role in maternity insurance fund income generation. In the short term, the contribution rate of the maternity insurance fund must be adjusted upward or the payment base expanded to receive additional contribution from all employees to avoid complete depletion of the fund. In the long term, we recommend the need to replenish the maternity insurance funds through proper investment options for the funds. We further recommend the need to look for other sources of funding social interventions based on existing practices in other countries.

## 1. Introduction

In crafting the Millennium Development Goals (MDGs), the “Millennium Fathers” recognised the need to accord the reproductive health of women a social responsibility rather than an individual one [1]. This is why MDG-5 demands global support for maternal health security. Since the early 1950s, China has consistently promoted new policies and programs to ensure high maternal survival rate in accordance with global best practices. Some of these notable interventions include the Instructions on the Implementation of the System of Public Medical Care for Staff Members of the People’s Governments at Various Levels, Political Parties, Mass Organizations and Public Institutions Attached to them. This policy was promulgated in 1952 and was followed by another policy called the Notice Concerning the Provisions on the Maternity Leave of Female Staff Members which was also promulgated in 1955 [2].

In 1995, China rejuvenated its maternal and child health laws to prioritize the allocation of national resources to improve maternal health quality services. In 2019, the comprehensive health reform policy vision (Healthy China Initiative 2030) was adopted by China. Inherent in this policy is a comprehensive national blueprint to protect maternal health. This policy provides path-breaking strategy to safeguard access to reliable healthcare for all citizens and especially women and children at all levels of social organization [3]. Today, village clinics, community health centers and urban hospitals have been restructured to support maternal health in a seamless and hierarchically organized three-tier structured network of the health services delivery system [4]. Since these interventions were rolled out, China has drastically reduced maternal mortality from 19.6 to 18.3 per 100,000 births. It has also helped China to become one of the few countries to have drastically reduced maternal mortality ratio in accordance with MDG-5.

In 1994, China’s Labor Department started the “Trial Measures on Maternity Insurance for Enterprise Staff and Workers” to help working mothers to obtain decent and affordable healthcare and livelihood before, during and after childbirth [5]. This policy was part of an overall objective to safeguard the legal rights and interests of female employees and provide them with basic allowance and medical care for qualified women. In response to public criticisms, the maternity health insurance scheme was reorganized in 1992 and extended into other provinces [6]. In 1995, however, major reforms were carried out, and the maternity insurance scheme became fully operational in all the provinces and autonomous regions of China [7]. In 2019, further reforms were done in line with the vision of the Healthy China Initiative to enable local councils to take control of the management of the maternity insurance scheme to ensure their effectiveness in providing reasonable allowance and proper care for women.

Over three decades, the maternity health insurance scheme has supported women across and this has helped reduced potential irretrievable catastrophic health consequences associated with pregnancy and childbirth [8]. The benefits of the maternity insurance scheme in China notwithstanding, it is not without challenges. In its current form, each employer is required to pay a percentage of a female employees’ wages or the national minimum wage (whichever is higher) to the locally-administered maternity insurance fund [9]. The percentage contribution ranges between 0.5% and 1.2% of the payment base, but the specific rate depends on each province. For example, in Zhejiang Province, employers’ contribution was reduced from 1.2%. Currently, employers are permitted to contribute between 0.8% and 1% of their employees’ salary or social wage. In Tianjin, the employer’s contribution to maternity insurance was reduced from 0.8% to 0.5% in 2015. In Jiangsu Province, different cities have different values. For example in Suzhou, the maternity insurance contribution rate is between 0.5% and 1%. Thus, the employers can elect to contribute any rate but not lower than 0.5%. The occasional reduction in the maternity insurance contribution rate is to encourage employers to employ women without feeling the burden of the additional maternity insurance levy [10].

However, reducing the contribution rate to encourage enrollment has negatively affected the income accrued into the maternity insurance fund without a corresponding increase in enrollment. In addition, the investment returns income from investing in the maternity insurance fund is extremely low. Most of the funds are invested in low-yielding government bills because of the need to avoid the high risk of loss of investment in high-yielding investment funds [11]. The limited fund inflow into maternity insurance is however inconsistent with the ever-increasing number of beneficiaries due to increased births. The persistence of this tendency is endangering the sustainability of funds across some provinces in China. As reported by Milcher [12], the maternity insurance scheme in some provinces such as Zhejiang, Anhui and Jiangsu started accumulating deficit in 2016 due to excess expenditure over income, and by 2018, the maternity insurance fund deficit had reached an unsustainable level.

In 2019, the maternity insurance fund balance in Guangxi Zhuang Autonomous Region also began accumulating escalating levels of deficits [13]. Even though this is not the first time that the maternity insurance scheme in these two regions has experienced a decline in fund balance, it is believed that the universal two-child policy program implemented across China in 2015 has contributed to the deficit accumulation of the maternity insurance fund.

If the current situation persists, it may lead to the depletion and possible collapse of the maternity insurance funds with devastating consequences for maternal health. Even though other factors contribute to fund depletion, the potential effect of the comprehensive two-child policy is much wider because the policy targets a higher population group and hence may have a bigger impact. There are suggestions to adjust the contribution rate of maternity insurance to ensure its sustainability [14].

The depletion of the maternity insurance fund in Guangxi Zhuang Autonomous Region has particularly attracted the concerns of policy analysts, policy-makers and researchers because the region is economically young among the regions in China. Thus, a weak maternity insurance scheme can affect economic development of the Guangxi Zhuang region at all levels. On the other hand, a decline in the maternity insurance scheme in Jiangsu Province (one of China’s most successful provinces) can cast doubts on the robustness of China’s social intervention policies [11].

This study sought to compare maternity insurance fund depletion in Jiangsu and Guangxi also because of the differences in how the maternity insurance scheme is managed and their repercussions on the sustainability of the maternity insurance funds. Firstly, the two regions have different levels of economic development. Their population characteristics are also different, and the maternity insurance scheme is better developed in Jiangsu Province than in the Guangxi Zhuang Autonomous Region. The two regions have different contribution rates, different payment rates and different demographic profiles which affect maternity insurance income and expenditure. Further, both Guangxi region and Jiangsu Province differ in terms of wage differences, unemployment, age differences, number of female employees in the formal sector and other predictors of the maternity insurance scheme.

Most importantly, the maternity insurance contribution and utilization rate in both provinces are differently regulated. While Guangxi Province has a fixed-contribution system across the province, the contribution rate of maternity insurance in Jiangsu is different from city to city. Employers in bigger cities such as Suzhou pay more than those in small cities. Another difference between the two provinces is that demographically, Guangxi Zhuang Autonomous Region has a high concentration of minority ethnic groups besides the Han majority ethnic group. Some of these include the Zhuangs (32%), the Pinghua, Hakka and Min, Galeo (0.4%), Dong (0.7%), Miao (1%) minorities and Vietnamese (0.6%) of the population. These minority groups were not affected by the restrictions of the one-child policy; hence the two-child policy should not change the rate of birth to affect maternity insurance [13].

Jiangsu Province is located in the mid-east of China, and it is the second-richest region in China in terms of GDP. Its proximity to Shanghai and large coastline make it an economically strategic area in China. These advantages have helped transform the province into a hotbed of economic activities with several flourishing industries. It currently ranks as the fifth most populous region in China and attracts more professionals from other parts of China to work in its major cities such as Nanjing, Kunshan, Suzhou, Changzhou, Wuxi, Zhenjiang, etc. Maternity insurance began in Jiangsu much earlier than in many other provinces with the onset of economic revolution in the early 1970s to attract more professional women into its industries and emerging academic institutions that needed professional women to expand its internationalization drive [15].

Management of Jiangsu’s maternity insurance scheme is considered one of the most effective in China and has become a reference point for other provinces. In 2015, for example, the insurance scheme paid all outstanding balances to healthcare facilities for all maternity insurance-related pre-natal examination fees, hospital delivery fees, family planning surgery fees, one-time nutrition subsidy fees and maternity allowance fees. On the other hand, the Guangxi Zhuang Autonomous Region (AR) is relatively an underdeveloped region, and its population is relatively poor. For this reason, a depleted maternity insurance scheme can widen the perceived inequity in maternal health among China’s provinces [16]. Additionally, a stable maternity insurance fund is necessary for Guangxi’s economic growth. In 2017, the government declared Guangxi Zhuang Autonomous Region as China’s official gateway to institute economic ties with the Association of Southeast Asian Nations (ASEAN) to rejuvenate the economic development of Guangxi [17]. This initiative can attract more young women (especially natives of Guangxi) to return and work in their home region after completing their education in major cities. Further, women constitute a substantial number of employees in the main export processing zones in Guangxi [18]. For example, the Beihai Export Processing Zone, Dongxing Border Economic Cooperation Area, Guilin National New and Hi-Tech Industrial Development Zone, Nanning Economic and Technological Development Area, Nanning National Hi-Tech Industrial Development Zone, Pingxiang Border Economic Cooperation Zone and the Yongning Economic Development Zone have more than 34% of employees as women that can potentially benefit from a maternity insurance fund [19]. The need to avoid the potential catastrophic health effect on women in both Jiangsu Province and the Guangxi Zhuang Autonomous Region should the maternal insurance scheme fail informed this study. The main research objectives included the following:

- To determine the effect of maternity insurance fund income factors on maternity insurance in Jiangsu Province and Guangxi Autonomous Region before and after the comprehensive two-child policy.
- To determine the effect of maternity insurance fund expenditure factors on maternity insurance depletion in Jiangsu Province and Guangxi Autonomous Region before and after the comprehensive two-child policy.
- To measure the impact of the additions to new births arising out of the comprehensive two-child policy on maternity insurance in the Jiangsu Province and the Guangxi Zhuang Autonomous Region.
- To determine the extent to which investment income from a maternity insurance scheme can be strengthened to increase inflow into the maternity insurance fund in both regions.
- To make policy recommendations to mitigate or eliminate the negative effect of the policy on maternity insurance funds in both provinces.

A brief literature review is presented before explaining the analytical model and data selection. The results are then presented and discussed. Finally, the limitations and suggestions for future research direction are discussed.

### Literature Review

China abolished the existing one-child population and family planning policy in 2015. This was replaced with a comprehensive or universal two-child policy. With the new policy, the government also abolished incentives for late parenthood and late marriage [20]. Subsequent to the revision of the population policy, proportional changes were made to marriage leave and incentives (paternity and maternity leave). The maternity insurance scheme in China is one of the social insurance funds designed to support the citizens. The rest are basic medical insurance, pension insurance, unemployment insurance and work-related injury insurance. In 2017, however, maternity insurance and basic medical insurance were combined into a single-spine insurance system to ensure a more egalitarian and sustainable development [21].

The management of maternity insurance in China has been decentralized; hence the day-to-day decision-making starts at the local community, county, provincial or regional government before it is approved at the national level [22]. As is with every form of insurance scheme, the maternity insurance scheme in China comprises of an inlet or inflow sources (income) and the outlet or outflow channels which are the expenditure component [23]. In most provinces in China, four main sources of funds flow into the maternity insurance fund. These sources include the maternity insurance contribution income, interest from the maternity insurance fund investment, voluntary contributions by enterprises and special allocations by the provincial government.

Currently, 97% of the maternity insurance fund income in most of the provinces in China comes from the contributions paid by employers for their female employees [24]. Per the law, only the employer contributes to the maternity insurance fund without proportional contribution from the employee as it pertains to the other social insurance funds. For this reason, the amount of money that is accrued in the maternity insurance scheme depends on the number of insured persons and the insurance contribution rate [25]. An increase in the female working population may lead to an increase in the enrollment rate. This may in turn increase the monetary inflow into the maternity insurance fund. Conversely, a reduction in female employment may affect the number of enrolled persons. This may also proportionally reduce the maternity insurance fund income [26].

Consistently, a rise or decline in the number of enrollments may correspondingly affect the expenditure pressure on the maternity insurance fund. This is without prejudice to the fact that not every enrolled person will utilize it.

An interpretation of the regulations that sets up the maternity insurance fund implies that the amount of funds accruing from the employer’s contribution also depends on the maternity insurance contribution rate and the payment base [27]. In order to ensure a fair contribution to the funds, the law that establishes the maternity insurance fund requires employers to pay a percentage of the female employees’ income or the existing basic or minimum wage depending on which one of them is higher. This percentage varies from province to province, but the regulation implies that an employer cannot contribute anything below the recommended percentage of the minimum or basic wage [3]. The reason for this instruction is that some female employees are paid far below the minimum wage in China. Therefore the regulation is intended to protect lower-income earners from being exploited by their employers in terms of maternity insurance contribution. Thus, by using the highest base to compute the contribution on behalf of their employees, the employer is inherently mandated to observe “utmost good faith” on behalf of their employees [28]. That notwithstanding, a lot of employers elect to pay the maternity insurance contribution at the lowest possible payment base even if the salary of their female employees is higher than the minimum wage [29]. Employers do this to avoid the burden of the charge. Unfortunately, the affected employees are afraid to insist on the right contribution largely because they are afraid to lose their job. In the work of [24], they explain that salaries and minimum wages themselves are also influenced by several external factors such as economic growth, seasonal changes, unemployment rate, age differences, geographical differences, elasticity of demand, wage equality and many other quantitative and qualitative factors.

Besides the payment base, there is also the contribution rate. Different provinces have different rates of contribution. Determining the contribution rate goes through several stages before acceptance. Firstly, a proposal is made by the municipal government of the sub-district based on the actual situation of the area. This initial proposal is further discussed, approved or adjusted by the provincial people’s government after which it is submitted to the State Council for human resources, social security and financial development for further deliberations and approval [30]. In the Guangxi Zhuang AR, the maternity insurance fund has been fixed at 0.5% of the payment base since 2014. This means that employers may elect to contribute at any rate but not lower than 0.5%. In Jiangsu, however, different cities and sub-districts have different rates. For example, the rate in Suzhou is higher than the rate in Dantu County. Currently, the rates range between 1% and 0.7% across provinces.

In China, local and provincial governments have been mandated to look for other feasible income sources to augment income flow into the fund, yet not much has been achieved in the last decade across provinces, municipalities and autonomous regions in this regard. Moreover, individual organizations are also encouraged to make voluntary contributions to the fund in order to ensure its sustainability. Cheng et al. [31], however, pointed out that in most provinces in China, many enterprises do not pay beyond the mandatory minimum contribution since it comes with no additional benefit such as tax exemption.

Three main variables determine the total fund expenditure across provinces. The first is the number of people that utilize the maternity insurance fund. Generally, not everyone who is enrolled and contributes to the fund actually utilizes the fund. It is only those who give birth in accordance with the family planning regulations and laws that qualify for the support. Even in this case, there are several qualified contributors who voluntarily refuse to utilize the funds for personal reasons. There are exemptions under both the one-child policy and the comprehensive two-child policy regulation. In both policies, certain groups of people were exempted from the limits on their births [32]. They can therefore give birth to additional children based on clearly spelt out qualifying requirements. For example, ethnic minorities and parents of disabled children have some form of exemptions if they applied for them. Even though this category of people is small, their decisions can potentially increase the expenditure burden of the maternity insurance fund [33].

The number of beneficiaries of the maternity insurance fund income is also controlled by external factors such as birth rate, death rate, the number of female employees in the formal sector and population policy, etc. For example, with the universal one-child policy, it is assumed that most of those qualified can utilize the insurance fund once in their lifetime. However, a change in population policy from one-child to the comprehensive two-child policy implies that women who hitherto could give birth to only one child can now give birth to more children. This equally implies that they can utilize the maternity insurance fund more often than under the universal one-child policy. The rate of utilization is not the same every year. The insurance fund experiences a higher rate of utilization in accordance with customs and beliefs in the Chinese zodiac. For example, in a typical dragon year, more births are recorded than other years hence a higher utilization rate. Reference [34] also explains that the impact of mothers who give birth to twins or triplets is marginal since such persons only receive a marginal increase in maternity insurance benefits which has very minimal impact on the maternity insurance fund balance.

The per capita utilization is the next factor that affects the iteration rate of the maternity insurance fund. This is the actual amount of money that is paid to each beneficiary who accesses the maternity insurance funds. In China, two systems of payment have been piloted in different regions and provinces. More recently, other provinces have introduced a hybrid system of payment based on different payment rates [35]. For example, in Jiangsu Province, the fixed-payment system of maternity insurance contribution is used to pay beneficiaries. In Jiangsu, the monthly payment made by the maternity insurance fund to a woman during their maternity leave is based on the average salary of women in the company. This means that a woman whose salary is low could receive more money during her maternity leave if she works at a company where other women receive a higher salary than her. In a similar manner, a woman whose salary is high can receive lower monthly support from the fund if most of the other contributors in the enterprise receive a very low salary [36]. The payment system in Guangxi Zhuang AR is a little different from that of Jiangsu Province due to certain disparities. The cost of pre-natal examination fee, delivery fee and family planning surgery fee is the same for all contributors. A minor adjustment is made on the one-time nutrition subsidy fee and maternity allowance for the period of leave based on specific circumstances [37]. These costs are determined by the local government and subsequently approved at the provincial and state level administration. Invariably the specific amount is determined by the level of economic development and cost of healthcare, inflation, etc.

## 2. Materials and Methods

### 2.1. Data Source

Some of the data were procured from the Statistical Year Books of Jiangsu Province and Guangxi Zhuang AR. These include data about the fund balance, number of insured individuals, utilization cost, wage of insured persons and number of beneficiaries. The maternity insurance contribution rate data came from the 2020 Social Insurance Rates in China issued by China Labour Watch. The birth rate, the death rate, the population of Jiangsu Province and Guangxi Zhuang Autonomous Region, minimum social wage, interest rate and the inflation rate in China were procured from Knoema Enterprise Data Solutions. This organization makes its data available to the public at no cost. To compare the pre-policy and post two-child policy period, nine years of data from 2011 to 2019 were collected. This period was selected because the first announcement of the decision of China to adopt the comprehensive two-child policy was made in 2011 even though the actual implementation started in 2015. This means that each province had up to four years to prepare for the change. By 2019, the new policy had been in operation for four years. Therefore, keeping 2011–2019 enables one to make a good comparison of the last four years before the policy and first four years after the policy. Moreover, four years after a change in policy is a reasonably good time to make an initial assessment of the impact of the policy. Table 1 shows the definition of variables and how they were measured.

### 2.2. Analytical Model

Panel data that contain variables obtained across the two provinces were assembled over even intervals in time and ordered chronologically. A panel data analysis method was employed in this research because panel data can help to model both the common and individual behaviors of maternity insurance fund behaviors in Jiangsu Province and Guangxi Zhuang Autonomous Region contemporaneously. Secondly, panel data were employed in this study because they contain more information, more variability and more efficiency than pure time-series data or cross-sectional data. The third reason for using panel data for this study is that they are capable of detecting and measuring statistical effects that cannot be detected and measured by pure time-series or cross-sectional data. Finally, we opted for panel data because they can help to reduce the estimation biases that may arise from aggregating groups into a single time series. We employed a series of data integrity tests to validate the quality of the data prior to the final analysis.

### 2.3. ADF Test of Stationarity

The ten-year data period is large enough to be cautious of unique disturbances that could lead to random shifts in the data within the period. Thus non-stationarity was conducted using the Augmented Dickey and Fuller (ADF) and the Phillips–Perron (PP) unit root tests. These two tests were applied because of the robustness of their inference and their strong reputation in the extant literature [38].

For example, the PP test is robust to test the general forms of heteroskedasticity in the error term $u_{t}$. The PP method was applied to test unit roots in the time-series data instead of the Augmented Dickey and Fuller (ADF) test because it accommodates small-sized samples as pertains in this case. The ADF also accommodates large and very complicated datasets. The generalized form of the ADF equation was applied to show deterministic specifications such as the intercepts, time, trends and number of lags. We fixed a significant value of 95% in the regression model. Appropriate graphs were used to inspect the structural breaks before deciding whether to test them or not. The Kwiatkowski–Phillips–Schmidt–Shin (KPSS) tes was used to establish the staionarity of the data [39]. We used Eviews 9 software to test the coefficient on the lagged variable.

### 2.4. Autoregressive Distributed Lag (ARDL) Model

If one of the data series is not integrated in the same order as the other variables, co-integration test may produce a spurious outcome due to a mismatch in the order of the variables. For this reason, the autoregressive distributed lag (ARDL) models test was used to test the degree to which the variables of interest co-integrated in the long run. The ARDL bound testing approach proposed by Paseran was applied to test the co-integration of the variables. This approach was used because it is efficient and consistent even in testing small data samples. As posited by [3], the maternity insurance fund balance is the dependent variable but the independent variables are divided into expenditure or outflow variables and income variables. The expenditure variables are the average wage of an insured person or minimum social wage, the number of insured persons and income from investment of maternity insurance fund. The income or inflow variables include the maternity insurance payment rate, the number of beneficiaries and average utilization cost. Our ARDL model is formulated mathematically as follows:

$$\begin{array}{c} \Delta MFB_{2t}=\beta_{0}+\sum_{i=1}^{k}\delta_{1}\Delta MPR_{t-i}\;+\sum_{i=1}^{k}\emptyset_{1}\Delta MIN_{t-i}+\sum_{i=1}^{k}\omega_{1}\Delta \;MAP_{t-i}\;+\\ \sum_{i=1}^{k}\gamma_{1}\Delta \;INT_{t-i}+\sum_{i=1}^{k}\varphi_{1}\Delta \;MAT_{t-i}+\sum_{i=1}^{k}\rho_{1}\Delta \;MEN_{t-i}+\lambda_{1}Nact_{t-i}\;+\\ \lambda_{1}INF_{t-i}+V_{t} \end{array}$$

where:

- $MFB_{it}$ is the maternity insurance fund balance for province *i* for time *t*;
- $MPR_{it}$ is the maternity insurance contribution rate for province *i* for time *t*;
- $MIN_{it}\;is\;the\;number\;of\;insured\;people$ for province *i* for time *t*;
- $MAP_{it}$ is the average wage of an insured person or minimum social wage for province *i* for time *t*;
- INT is the interest rate of the maternity insurance fund investment;
- $MAT_{it}$ is average utilization cost for province *i* for time *t*;
- $MEN_{it}$ is the number of beneficiaries in a year for province *i* for time *t*;
- $Nact_{it}$ is the addition to the beneficiaries as a result of the two-child policy for province *i* for time *t*;
- INF is the inflation rate in China.

We included a new construct called addition to the insured population as a result of new births arising out of the universal two-child policy. We adopted the computation proposed by [11] and previously used in [40]. This model determines new additions to the insured population as a result of the new population policy as follows:

(1) $$Nact_{t}={\left(NCT\right)}_{t}\times \frac{{\left(MIN\right)}_{t}}{{\left(TP\right)}_{t}}$$

where ${\left(Nact\right)}_{t}$ represents new additions to the insured population of the region or province in year *t* resulting from the two-child policy. In this case, ${\left(NCT\right)}_{t}$ represents new additions to the population of the region or province in year t resulting from the two-child policy, ${\left(TP\right)}_{t}$ represents the total population of the region or province in year *t*, and MIN represents the number of insured persons in the region or province. Zhang’s [11] model of computing the increment in maternity insurance beneficiaries arising from the maternity insurance scheme was chosen because it accounts for changes in birth rate, death rate and the probability of an insured person giving birth to twins (1/89) [11].

To model the long-run relationship for the ARDL equation, it is necessary to determine an appropriate lag length. An extremely short lag length may lead to incorrect specification, but an extremely long lag length can affect the degrees of freedom [41]. We strictly applied the rule of thumb that says that the maximum lag for annual data should be either 1 or 2 [42]. We used maximum criteria of 2 and the Akaike Information Criteria (AIC) to select the optimum lag length. The AIC are based on a high likelihood value. Finally, the serial independence of the data series was checked using 3 residual diagnostic tests, namely Ramsey RESET Test, Breusch–Godfrey Serial Correlation LM Test and Breusch–Pagan–Godfrey Heteroskedasticity Test as our residual diagnostic tests after which we conducted a bounds test.

### 2.5. VECM Granger Causality Test

Subsequent to evaluating the long-run relationship between the variables of interest, we applied the Granger causality test to establish causality between the variables. As the results show co-integration between the series, a Vector Error Correction Model (VECM) was estimated as follows:

$$\left(1-L\right)=\left[\begin{array}{c} \begin{array}{c} MFB_{1t}\\ MPR_{t}\\ MIN_{t}\\ MAPt\\ INT_{t}\\ MAT_{t}\\ MEN_{t} \end{array}\\ Nact_{t}\\ INF_{t} \end{array}\right]+\sum_{i=1}^{p}\left(1-L\right)\left[\begin{array}{lllllllll} d_{11i} & d_{12i} & d_{13i} & d_{14i} & d_{15i} & d_{16i} & d_{17i} & d_{18i} & d_{19i}\\ d_{21i} & d_{22i} & d_{23i} & d_{24i} & d_{25i} & d_{26i} & d_{27i} & d_{28i} & d_{29i}\\ d_{31i} & d_{32i} & d_{33i} & d_{34i} & d_{35i} & d_{36i} & d_{37i} & d_{38i} & d_{39i}\\ d_{41i} & d_{42i} & d_{43i} & d_{44i} & d_{45i} & d_{46i} & d_{47i} & d_{48i} & d_{49i}\\ d_{51i} & d_{52i} & d_{53i} & d_{54i} & d_{55i} & d_{56i} & d_{57i} & d_{58i} & d_{59i}\\ d_{61i} & d_{62i} & d_{63i} & d_{64i} & d_{65i} & d_{66i} & d_{67i} & d_{68i} & d_{69i}\\ d_{71i} & d_{72i} & d_{73i} & d_{74i} & d_{75i} & d_{76i} & d_{77i} & d_{78i} & d_{79i}\\ d_{81i} & d_{82i} & d_{83i} & d_{84i} & d_{85i} & d_{86i} & d_{87i} & d_{88i} & d_{89i}\\ d_{91i} & d_{92i} & d_{93i} & d_{94i} & d_{95i} & d_{96i} & d_{97i} & d_{98i} & d_{99i} \end{array}\right]\left[\begin{array}{c} \begin{array}{c} MFB_{1t-i}\\ MPR_{t-i}\\ MIN_{t-i}\\ MAP_{t-i}\\ INT_{t-i}\\ MAT_{t-i}\\ MEN_{t-i} \end{array}\\ Nact_{t-i}\\ INF_{t-i} \end{array}\right]+\left[\begin{array}{c} \begin{array}{c} \lambda_{1}\\ \lambda_{2}\\ \lambda_{3}\\ \lambda_{4}\\ \lambda_{5}\\ \lambda_{6}\\ \lambda_{7} \end{array}\\ \lambda_{8}\\ \lambda_{9} \end{array}\right]\left[ECM_{t-1}\right]$$

This (1 − *L*), the lag operator, denotes the number of lags in the model; and the $ECM_{t-1}\;$ denotes the error correction term. Following the test of causality, more inferential statistical analyses were conducted using linear function to establish the strength of causality between the dependent variable (maternity fund balance) and the eight independent variables.

## 3. Results

Table 2 presents the descriptive overview of the variable items for both provinces. Since the two of the provinces are within the same country, interest rates and inflation rates for the period barely differed from each other. The mean maternity insurance contribution rate differs between the provinces because of different rates charged in Jiangsu. For example, the rate in Suzhou, Kunshan, Nanjing, Wuxi, Changzhou, Zhenjiang and the major cities is higher than the case in small towns or communities. In the case of the Guangxi Zhuang Autonomous Region, contribution rate has been pegged at 0.5% for a considerably long period of time. The skewness and kurtosis values are within the acceptable ranges of normality.

The table also shows differences between Jiangsu Province and Guangxi with regards to the mean values of the number of insured people (2480 and 2182), the average wage/minimum wage (1089 and 958.32), number of beneficiaries (72.38 and 63.69) and utilization cost (1.88 and 1.66). In these cases, the values in Jiangsu Province are higher than in Guangxi Zhuang Autonomous Region. Since these variables are largely influenced by the size of the population and economic growth, it is not surprising because Jiangsu is far bigger in terms of population and economic development and hence has a high standard of living. The mean values of inflation (4.97) and interest accrued from investment (3.85) in both areas are similar because they invest in the same government-sponsored securities. In all these cases, the standard deviations, skewness and kurtosis levels are within an acceptable range of normality and are higher in the case of Jiangsu Province than in Guangxi Province.

Table 3 presents the results of the ADF, PP and KPSS tests. The results of the ADF and the PP affirm the null hypothesis that the time series is integrated of order one. At a 95% confidence interval, the *t*-test statistic value for each variable is greater than their respective critical values. The same trend was observed regarding the KPSS values. In terms of stationarity, the combined effect of the three results is a sufficient basis to reject the null hypothesis that the series is stationary at level. Again, the results of the first difference analysis support this case. This is because at first difference, the *t*-statistics values are lower than the critical values for each variable, and this further supports the non-stationarity of the time series at level but stationary at first differences.

### 3.1. Co-Integration Analysis

In a co-integration test, all the series must be integrated in the same order, but the results of our KPSS tests of stationarity show that one of the series of data is integrated at 1(0) and others at 1(1). To avoid spurious co-integration results, Pesaran et al.’s ARDL model that uses a bound test approach to co-integration was used to verify the short- and long-run relationship among the time-series data [43]. Model 1 in Table 4 presents the results of the ARDL. The table shows that if maternity insurance fund balance (MFB) is treated as the dependent variable while contribution rate, number of insured people, average wage of an insured person or minimum social wage, interest accrued from the maternity insurance fund investment, average utilization cost, the number of beneficiaries, the addition to the beneficiaries and the inflation rate are treated as independent variables, the separate F-statistics values are bigger than the upper bound values at 2.5% significant value. At 1% significant value, the respective F-statistics values fall in-between the lower and upper bound. This means that there is co-integration between each of the independent variables and the dependent variable. Model 2, on the other hand, presents the results of a residual diagnostic test based on the Breusch–Pagan–Godfrey Heteroskedasticity Test and the Breusch–Godfrey Serial Correlation LM Test. The F test scores and *p* values of the Breusch–Godfrey Serial Correlation LM Test indicate that there is no serial correlation among the variables. The scores of the Breusch–Pagan–Godfrey Heteroskedasticity Test also confirm the absence of a heteroscedasticity problem. This is in addition to the good score of the Ramsey RESET Test of model stability that shows that the two models are dynamically stable. These results therefore confirm high validity of the model for the analysis, and further analysis can be conducted.

Table 5, on the other hand, presents the results of the long- and short-run impact of the variables on maternity insurance fund balance in both provinces. Their statistical significances were also included to determine the importance of their impact. Consistent with the literature, the impact of maternity insurance income variables (contribution rate, number of insured people, wages and interest accrued) is positive and statistically significant both in the short and long term. The results, however, indicate that the impact of maternity insurance contribution rate (MAR) to the maternity insurance fund decays with time, giving a long-run limited impact in both provinces. Thus the positive impact is stronger in the short term, but in the long term, its influence or contribution to the stability of the funds reduces. On the other hand, the positive impact of interest from investment (INT) on the maternity insurance fund is insignificant in both provinces, giving a major cause for concern on its importance in maternity insurance fund income generation.

This is confirmed by the fact that in the long run, the impact is highly negligible altogether. The short- and long-term impact of maternity insurance payment base (MAP) and the number of insured people (MIN) is greater in Jiangsu than in the Guangxi area, but their effects on income flow into the maternity insurance fund are individually significant. The other side of the analysis shows the impact of the maternity insurance expenditure indicators on the maternity insurance fund. For example, the average utilization cost (MAT) and the number of beneficiaries (MEN) have negative and statistically significant impact (long and short term) on the maternity insurance fund in both Jiangsu Province and the Guangxi Zhuang Autonomous Region. However, it is evident that the average maternity insurance utilization cost (MAT) in Jiangsu Province exerts a higher impact on maternity insurance expenditure and fund balance in Jiangsu than in Guangxi. This discrepancy may be explained by the fact that the pre-natal examination fee, hospital delivery fee, family planning surgery fee, one-time nutrition subsidy fee and maternity allowance fee that constitute this component of maternity expenditure are much higher in Jiangsu than in Guangxi. Moreover, it is also not surprising that the number of beneficiaries (MEN) of the maternity insurance fund exerts higher impact on maternity insurance income in Jiangsu than in the case of Guangxi. Besides the high population in Jiangsu, the percentage of the female working population in the two provinces is different, and Jiangsu province has more than Guangxi.

It is equally significant to note that the addition of new births (Nact) as a result of the comprehensive two-child policy is also negative and statistically significant. The impact of the addition to the beneficiaries as a result of the two-child policy on the maternity insurance fund balance is a very important observation in this study. As noted in the table, the impact worsens with time because the effect is stronger in the long term than in the short term which is a reason to look for long-term solutions in addition to short-term ones. In this case, the impact is more strongly felt in the Guangxi Zhuang Autonomous Region than in the Jiangsu Province in the long term. Even though the effect of inflation (INF) on maternity insurance in both provinces is not significant in the short term, the negative effect on fund balance is significant in the long term in both provinces. The insignificant impact of inflation on the maternity insurance fund balance in both provinces implies that laudable evidence of China’s strong economic management has culminated in an overall low level of inflation across industries. The long-term result of the impact of inflation, however, is an indication that the current situation of the minimal or insignificant impact of inflation on the maternal insurance fund may not be permanent. Since this is outside the control of the administrators of the scheme, it adds to the growing evidence looking for alternative sources of income that can offset any future effect of high inflationary rate on the maternity insurance fund balance. This may include looking for high-interest-yielding portfolios in the securities market.

### 3.2. Granger Causality Test

Finally, the result of the VECM Granger causality test of the relationships between the variables is presented in Table 6 (Jiangsu Province) and Table 7 (Guangxi Zhuang AR). As co-integration was confirmed by the preliminary analysis, we expected a unidirectional or bi-directional causality among the series. In the case of Jiangsu Province, the table shows significant uni-directional causality from maternity insurance contribution rate for time, number of insured people, wages and interest accrued in the maternity insurance fund. The lagged error term in the table is negative and significant at a 5% confidence level. This implies that the speed of adjustment toward log run equilibrium has a negative sign which ensures that any deviation in one direction is automatically readjusted back to equilibrium. This observation holds true for both Jiangsu Province and Guangxi Zhuang AR. Similarly, the results support the hypothesis that average utilization cost, the number of beneficiaries, the addition to the beneficiaries as a result of the two-child policy and the inflation rate in China Granger causes depletion of the maternity insurance scheme in both Jiangsu and Guangxi. In the case of the two regions, the results also show that the effects of the variables are valid both in the short term and in the long term in both regions.

## 4. Discussion

The comprehensive or universal two-child policy was introduced in China in 2015 to correct some of the challenges the country faced with the existing one-child family planning and population policy. One of the objectives of implementing the universal two-child policy was to slow down the growing burden of dependency ratio that comes with a soaring aging population. It has been approximately five years since the policy was introduced, and some of its successes and challenges are beginning to emerge. The extant literature shows that the after-effect of the universal two-child policy on population boom has not been as drastic as envisaged prior to its introduction. The results of this study, however, suggest the need to reorganize maternity insurance fund in both Jiangsu and Guangxi Provinces to avert their imminent collapse due to excessive deficit accumulation. Three critical issues were revealed by the findings of this research. In both Jiangsu Province (an economically developed province) and Guangxi Zhuang Autonomous Region (a least-developed region), the impact of the addition to new birth as a result of introducing the universal two-child policy is significant since women can give birth to more children and are utilizing the funds more often than pertained under the one-child policy.

The results also indicate that the impact of maternity insurance contribution rate to the maternity insurance fund decays with time giving a long-run limited impact on fund flow in both provinces. Thus the positive impact is stronger in the short term, but in the long term, its influence or contribution to the stability of the funds reduces. Currently, the two different systems of setting the contribution rate in both Jiangsu Province and Guangxi Zhuang Autonomous Region have not brought the needed income to offset the impact of the addition to new births. In the short term, since an increase in the contribution rate can cause apprehension among employers, as well as affect female employment, it is suggested that the contribution policy needs to be expanded. Every employee is a potential mother or father and hence must be made to contribute to the maternity insurance fund just like with the pension funds. If the payment base is wider, the rate of contribution can even be reduced further down with minimal impact on the fund balance. This should be the case with Guangxi Zhuang AR in particular since it has the lowest contribution rate so far. The effect of the addition to new birth on maternity insurance fund depletion may grow stronger in the long term in Guangxi since the region already has a large number of minority ethnic groups (Zhuangs, Pinghua, Hakka and Min, Galeo, Dong Miao) who have never been restricted in terms of birth. The opportunity given to the majority ethnic group to give birth to more than one can compound the problem in a region where the contribution base is lower due to poor economic development. This observation is consistent with the earlier work of [11] who indicated that with the current contribution rate, the entire maternity insurance fund in most provinces in China may be completely depleted by the end of 2021.

In the long term, a more robust funding system must be put in place because the contribution rates cannot be increased arbitrarily without consequences for female unemployment or underpayment. Since employers pay the larger proportion of the premium, they are more likely to reduce the cost burden in recruiting a female employee. This may further compound the already socio-economic imbalances between males and females in the areas.

One area that can be rejuvenated is income from investment. The results show that the positive impact of interest from investment in the maternity insurance fund is, however, insignificant in both provinces, giving a major cause for concern on its role in maternity insurance fund income generation. The fund managers must invest the maternity insurance fund in high-yielding securities in order to become an important component of income for the fund. China can learn from other countries of Canada, United Kingdom, USA and Tanzania where effective investment strategies in the bond market, real estate and other high-yielding securities are being used. These portfolios guarantee immediate and high returns to cover expenses and future cash needs of the insurance funds, and this can shore up insurance funds’ income. Even in China, private insurance companies that emerged within the last decade offer higher services to their clients because they invest in higher-yielding options without having to increase the contribution rate arbitrarily. This explains the growing private healthcare plan market in China, and the state-led maternity insurance fund managers can learn from them. Suffice to say that such an ambitious venture may come at a cost, but this can be controlled [23].

For example, China’s hedge funds are good destinations for maternity insurance funds. It has seemingly drawn to prospects of superior returns with insignificant increases in systematic risk. It has proven to be the “game-changer” for investors that lost confidence in China’s economy after the devastating effect of the coronavirus pandemic. The hedge funds have developed a systematic and well-regulated financial partnership between the state and government in which pooled funds and regulated employer strategies are being used to earn active returns for their investors. China can learn from how social health insurance programs in other countries are supported with sustainable private-public supported funding schemes. For example, in Ghana, the national health insurance is supported by a 1% levy on fuel products. China can learn from this endeavor to secure better maternal health security for women in Guangxi.

Finally, China must learn from its previous successes in funding health priority health policies such as Health China 2030 Initiative. These have well-structured and sustainable funding schemes. China is already well noted for effectively managing large-scale social intervention programs or initiatives with a larger funding base such as the ecological civilization and beautiful China dream, the Belt and Road Initiative, etc. Maternity insurance can benefit from such elaborate funding opportunities if there is the political will to do so. Such an initiative can further bolster China’s current global leadership in promoting maternal health toward the attainment of the fifth Millennium Development Goal [23]. This is more important for Jiangsu and Guangxi Zhuang Autonomous Region because it will further demonstrate China’s commitment to creating a future with shared value and prosperity for both majority and minority ethnic groups.

## 5. Conclusions

The key contribution of this paper is the fact that the addition of new births arising out of the introduction of the universal two-child policy in China has led to depletion in the funds in the maternity insurance scheme in some provinces in China. In this study, the data on maternity insurance income and expenditure in two regions, namely Jiangsu Province and Guangxi Zhuang Autonomous Region, were analyzed. These two provinces differ in terms of economic development, demographic composition and structure of the management of the maternity insurance scheme, but the trend in the effect of the new policy on the maternity insurance scheme is almost the same. The paper suggests the need to boost income through a more sustainable contribution rate, investment returns and reliable source of additional income. We believe that strengthening maternity insurance in Guangxi Zhuang AR in particular, which is home to minority ethnic groups and other poorer farmers, will consolidate China’s determination to safeguard the health of minorities and other less endowed rural communities in the greatest possible way. This will further consolidate China’s strong progress toward the attainment of health-related Millennium Development Goals.

There are, however, some limitations of this paper. The data for this research span only a period of nine years between 2011 and 2019. If a longer range of data had been selected, it may have revealed some significant trends that are not revealed in this paper. Secondly, there are many external factors that also affect the variables that were empanelled for this study. Even the measurement of these variables is not without dispute. This limits the generalisability of the findings of this research. This research also suffers from secondary data bias. Not all data on maternity insurance in China could be validated from another independent and international database. Thus the results and findings are relevant to the extent of accuracy of the publicly available data on maternity insurance used in this research. We request future research that uses novel and more robust analytical techniques to reveal other patterns. For example, a systems dynamic model can help to conduct more accurate sensitive analysis. We call for the development of optimization models that can help predict or determine the optimal rate of maternity insurance contribution that must be instituted across China to maintain a viable maternity insurance scheme. Even in advanced economies, the most effective government policies do not necessarily generate the envisioned results as is the case of the universal two-child policy. Therefore, future research must also look at other unintended social consequences of the universal two-child policy in China and how it can be overcome.

## Figures and Tables

**Table 1:** Definition of variables and measurement scale.

Number	Name of Variable	Meaning	Variable Expressions and Assignments	Unit of Measurement
1	Birth rate	Births	0.00937	Rate
2	Births	Number of Births	The number of insured*Birth rate	Person/year
3	Death rate	Mortality	0.007	Rate
4	Deaths	Number of Deaths	The number of insured*Death rate	Person/year
5	MAT	People Who Enjoy Treatment	Births-Births*0.011236 + Second child comprehensive	People
6	INF	Inflationary Rate	Inflation rate	Rate
7	MFB	Income of Maternity Insurance Fund Balance	Maternity insurance income LESS xpenditure	RMB/year
8	INT	Interest Accrued to Fund	Interest rate	Rate
9	NCT	Number of New Insured per Year	New insured people = WITH LOOKUP (Time,([(2017,0)-(2027,4 × 10^−6^)],(2017,339040),(2018,339040),(2019,339040),(2020,339040),(2021,339040),(2022,339040),(2023,339040),(2024,339040),(2025,339040),(2026,339040),(2027,339040)))	Persons
10	MAP	Average Wage	Average wage/Minimum wage	RMB
11	MPR	Contribution Rate	Range between 0.05% and 0.1%	Rate
12	MIN	Number of Insured Persons	INTEG (Births-Deaths + New insured people, 1.51032 × 10^−7^)	Person/year

**Table 2:** Descriptive statistics of variables.

	Jiangsu Province								Guangxi Zhuang AR							
	MPR	MIN	MAP	INT	MAT	MEN	Nact	INF	MPR	MIN	MAP	INT	MAT	MEN	Nact	INF
Mean	5.00	2480.00	1089.00	3.85	1.88	72.38	2.68	4.97	0.50	2182.40	958.32	3.85	1.66	63.69	1.66	4.97
Maximum	8.00	17,654.00	2480.00	5.77	7.44	511.46	9.72	24.24	1.00	15,535.52	2182.40	5.77	6.54	450.08	6.54	24.24
Minimum	5.00	999.00	428.00	3.85	0.37	4.49	0.39	−1.40	0.50	873.12	376.64	3.85	0.33	3.95	0.33	−1.40
Std. Dev.	0.17	24.49	13.42	3.21	1.44	107.95	5.06	6.34	0.17	21.55	11.81	3.21	1.27	95.00	1.27	6.34
Skewness	−0.37	1.86	−0.23	1.09	2.50	−1.51	1.73	1.78	−0.37	1.64	−0.20	1.09	2.20	−1.33	2.20	1.78
Kurtosis	3.00	3.01	5.20	1.82	1.01	3.19	2.09	2.23	0.12	4.23	3.91	1.82	2.24	5.07	1.98	2.23
Obs	9	9	9	9	9	9	9	9	9	9	9	9	9	9	9	9

**Table 3:** Results of non-stationarity and staionarity tests for whole data.

ADF Unit Root Test			PP Unit Root Test		KPSS Unit Root Test	
Variable	*t*-Statistics	Critical Value	*t*-Statistics	Critical Value	LM-Statistics	Critical Value
Level						
MIN	−0.34638	−2.981038	−0.37271	−2.891607	0.764397	0.463
MAP	−0.70593	2.981038	−0.706	−2.831986	0.732981	0.463
INT	−2.24627	2.981038	−0.50178	−2.772365	0.643292	0.463
MAT	−0.6762	2.981038	−0.67628	−2.712745	0.853935	0.463
MEN	−0.66134	2.981038	−0.66141	−2.653124	0.532865	0.463
Nact	−2.10135	2.981038	−0.46941	−2.593503	0.863949	0.463
INF	−2.05304	2.981038	−0.45862	−2.533882	0.753842	0.463
MPR	−346932	2.981038	−0.52986	−2.474262	0.853275	0.463
MFB	−1.54962	2.981038	−1.66075	−2.414641	0.3854401	0.463
First Difference						
MIN	−2.91472	2.981038	−2.91472	−2.448705	0.532168	0.463
MAP	−4.0226	2.981038	−4.04253	−2.508429	0.829521	0.463
INT	−2.07719	2.981038	−0.46401	−2.563693	0.606423	0.463
MAT	−0.65391	2.981038	−0.65398	−2.623313	0.805259	0.463
MEN	−0.66877	2.981038	−0.66884	−2.682934	0.853290	0.463
Nact	−2.8587	2.981038	−2.93352	−2.747327	0.2722206	0.463
INF	−2.92085	2.981038	−2.99729	−2.807052	0.2781385	0.463
MPR	−3.54596	2.981038	−3.54596	−2.866776	0.2933616	0.463
MFB	−3.06162	2.981038	−4.19128	−2.866776	0.0799546	0.463

**Table 4:** Results of bound test for whole data.

**Model 1**			**Results of Bound Test for Whole Data**					
**Critical Value Bounds**			**Diagnostic Tests**					
**Significance**	**I (0) Bound**	**I (1) Bound**	**Serial Correlation LM Test**		**Heteroskedasticity Test**		**Ramsey RESET Test**	
10%	5.59	6.26	*F-statistic*	*Prob.*	*F-statistic*	*Prob.*	*F-statistic*	*Prob.*
5%	6.56	7.3	0.8647	0.43	0.72321	0.5858	2.3572	0.1218
2.50%	7.46	8.27						
1%	8.74	9.63						
*F-Statistic*	9.26							
**Model 2**			**Bound Test for Whole Data**					
**Critical Value Bounds**			**Diagnostic Tests**					
**Significance**	**I (0) Bound**	**I (1) Bound**	**Serial Correlation LM Test**		**Heteroskedasticity Test**		**Ramsey RESET Test**	
10%	2.72	3.77	*F-statistic*	*Prob.*	*F-statistic*	*Prob.*	*F-statistic*	*F-statistic*
5%	3.23	4.35	0.6582	0.76	2.31859	0.13	2.0836	0.15
2.50%	3.69	4.89						
1%	4.29	5.61						
*F-Statistic*	7.48							

**Table 5:** Long- and short-run analysis.

	Long Run		Short Run	
	Jiangsu	Guangxi	Jiangsu	Guangxi
Constant	1.2609 **	1.0238 **	1.5107 **	2.3484 **
	(0.03)	(0.02)	(0.05)	(0.04)
MPR	0.5278 **	0.3470 **	0.5816 ***	0.4037 **
	(0.02)	(0.05)	(0.00)	(0.03)
MIN	0.5278 ***	0.6400 **	0.2861 **	0.4047 ***
	(0.00)	(0.04)	(0.04)	(0.00)
MAP	0.2804 ***	0.1843 **	0.0505 **	0.0908 **
	(0.00)	(0.02)	(0.00)	(0.01)
INT	0.0063	0.0003	0.0789	0.0659
	(0.84)	(0.72)	(0.83)	(0.94)
MAT	−0.0875 ***	−0.0356 ***	−0.0445 ***	−0.0143 ***
	(0.00)	(0.00)	(0.00)	(0.00)
MEN	−0.6192 **	−0.5309 ***	−0.0410 ***	−0.0080 ***
	(0.04)	(0.00)	(0.00)	(0.00)
Nact	−0.3082 **	−0.3902 ***	−0.0392 ***	−0.0936 ***
	(0.03)	(0.00)	(0.01)	(0.01)
INF	−0.1931 ***	−0.1493 ***	−0.0312 ***	−0.0423 ***
	(0.00)	(0.00)	(0.00)	(0.00)

*** Significant at 99% Confidence Level, ** Significant at 95% Confidence Level.

**Table 6:** Vector Error Correction Model (VECM) Granger causality analysis (Jiangsu Province).

Variable	Dependent				Short-Run Causality					Long-Run Causality
Independent	ΔNFB	ΔMIN	ΔMAP	ΔINT	ΔMAT	ΔMEN	ΔNact	ΔINF	ΔMPR	ΔECT
ΔNFB	-	0.432 (0.01)	1.931 (0.03)	1.31 (0.012)	0.023 (0.00)	0.094 (0.04)	0.003 (0.037)	0.094 (0.04)	0.642 (0.043)	−0.234 [0.403] (0.001)
ΔMIN	0.235 (0.002)	-	0.286 (0.00)	1.342 (0.02)	0.321 (0.02)	0.324 (0.50)	1.030 (0.01)	0.207 (0.04)	1.432 (0.03)	0.591 [0.632] (0.000)
ΔMAP	1.053 (0.05)	1.034 (0.01)	-	1.049 (0.02)	1.034 (0.35)	0.104 (0.03)	0.320 (0.10)	0.383 (0.03)	0.321 (0.04)	0.492 [0.538] (0.004)
ΔINT	2.318 (0.001)	2.832 (0.02)	2.731 (0.01)	-	1.932 (0.00)	0.321 (0.031)	1.832 (0.00)	0.983 (0.01)	1.492 (0.00)	0.002 [0.739] (0.022)
ΔMAT	0.217 (0.012)	0.739 (0.00)	0.921 (0.01)	0.732 (0.00)	-	1.044 (0.01)	0.320 (0.11)	0.534 (0.02)	0.348 (0.03)	−0.641 [0.392] (0.003)
ΔMEN	0.243 (0.35)	2.620 (0.03)	1.004 (0.03)	0.032 (0.30)	0.046 (0.03)	-	0.491 (0.00)	0.591 (0.03)	0.578 (0.02)	−0.713 [0.649] (0.050)
ΔNact	1.830 (0.022)	3.831 (0.012)	2.832 (0.01)	0.231 (0.010)	0.821 (0.001)	0.321 (0.031)	-	0.273 (0.00)	1.234 (0.00)	−0.632 [0.903] (0.036)
ΔINF	0.920 (0.03)	2.043 (0.05)	1.027 (0.03)	1.003 (0.30)	0.004 (0.05)	0.012 (0.05)	0.930 (0.00)	-	0.284 (0.03)	−0.431 [0.459] (0.058)
ΔMPR	0.075 (0.00)	0.008 (0.01)	2.041 (0.00)	0.005 (0.01)	1.007 (0.001)	1.032 (0.003)	0.532 (0.02)	1.043 (0.00)	-	−0.739 [0.0423] (0.005)

**Table 7:** VECM Granger causality analysis (Guangxi Zhuang Autonomous Region).

Variable	Dependent				Short-Run Causality					Long-Run Causality
Independent	ΔNFB	ΔMIN	ΔMAP	ΔINT	ΔMAT	ΔMEN	ΔNact	ΔINF	ΔMPR	ΔECT
ΔNFB	-	0.392 (0.04)	0.834 (0.00)	0.462 (0.02)	0.619 (0.05)	0.638 (0.031)	0.953 (0.00)	0.549 (0.01)	0.932 (0.00)	0.421 [0.318] (0.003)
ΔMIN	0.642 (0.01)	-	0.423 (0.01)	0.043 (0.02)	0.842 (0.00)	0.830 (0.03)	0.743 (0.00)	0.853 (0.01)	0.618 (0.00)	0.442 [0.713] (0.012)
ΔMAP	0.361 (0.05)	0.830 (0.04)	-	0.639 (0.00)	1.732 (0.05)	1.281 (0.00)	0.849 (0.00)	0.642 (0.03)	1.762 (0.02)	0.391 [0.502] (0.002)
ΔINT	0.842 (0.02)	0.732 (0.04)	0.382 (0.00)	-	0.303 (0.02)	0.639 (0.03)	0.482 (0.04)	0.713 (0.03)	0.851 (0.00)	0.019 [0.623] (0.048)
ΔMAT	0.642 (0.01)	0.743 (0.01)	0.734 (0.043)	0.593 (0.00)	-	0.371 (0.00)	0.842 (0.03)	0.641 (0.048)	1.031 (0.03)	−0.486 [0.835] (0.001)
ΔMEN	0.034 (0.05)	1.462 (0.00)	0.451 (0.00)	0.834 (0.00)	0.673 (0.02)	-	0.632 (0.01)	0.842 (0.04)	0.841 (0.01)	−0.303 [0.492] (0.002)
ΔNact	0.741 (0.00)	0.532 (0.01)	0.932 (0.00)	0.632 (0.00)	0.632 (0.03)	0.742 (0.00)	-	0.672 (0.01)	0.741 (0.01)	−0.494 [0.412] (0.030)
ΔINF	0.819 (0.01)	0.084 (0.00)	0.482 (0.02)	0.733 (0.00)	1.173 (0.00)	0.531 (0.00)	0.749 (0.03)	-	0.734 (0.00)	−0.494 [0.931] (0.032)
ΔMPR	0.093 (0.02)	0.026 (0.03)	0.382 (0.05)	0.184 (0.05)	0.841 (0.03)	0.842 (0.03)	0.853 (0.02)	1.732 (0.01)	-	−0.592 [0.094] (0.001)

## Data Availability

The data for this research are held by the authors and will be made available upon reasonable request.
